# The role of L-Arginine metabolism in the hallmarks of cancer as an emerging pathway to drug design

**DOI:** 10.3389/fphar.2026.1851940

**Published:** 2026-05-22

**Authors:** Gael Garcia-Valdez, Angel Emiliano Vazquez-Galvan, Jose Luis Gonzalez-Llerena, Daniela Treviño-Almaguer, Daniela Liliana Gallegos-Ibarra, Daniela Guajardo-Gonzalez, Isaias Balderas-Renteria, Maria del Rayo Camacho-Corona, Bryan Alejandro Espinosa-Rodriguez

**Affiliations:** 1 School of Chemistry, Laboratory of Biopharmacy, Universidad Autonoma de Nuevo Leon, San Nicolas de los Garza, Nuevo Leon, Mexico; 2 Faculty of Public Health and Nutrition, Universidad Autonoma de Nuevo Leon, Monterrey, Nuevo Leon, Mexico; 3 School of Chemistry, Laboratory of Molecular Pharmacology and Biological Models, Universidad Autonoma de Nuevo Leon, Monterrey, Nuevo Leon, Mexico; 4 School of Chemistry, Laboratory of Pharmaceutical Chemistry, Universidad Autonoma de Nuevo Leon, Monterrey, Nuevo Leon, Mexico

**Keywords:** cancer metabolism, L-arginine auxotrophy, L-arginine metabolism, metabolic reprogramming, tumor microenvironment

## Abstract

Cancer remains a leading cause of mortality worldwide and continues to challenge therapy due to its biological complexity and metabolic plasticity. Among the pathways supporting malignant progression, L-arginine metabolism has emerged as a key regulator of tumor cell fitness, integrating nutrient sensing, bioenergetics, redox balance, and immune modulation. In cancer, arginine homeostasis is frequently rewired, generating L-arginine auxotrophy and increased dependence on extracellular L-arginine, which contributes to several hallmarks of cancer, including proliferation, metabolic adaptation, angiogenesis, immune evasion, and tumor aggressiveness. This review discusses the molecular mechanisms through which L-arginine metabolism reshapes tumor biology, focusing on signaling pathways such as mTORC1 and on tumor-immune interactions within the tumor microenvironment. Therapeutic strategies targeting this pathway, including L-arginine deprivation therapies and emerging small-molecule modulators, are also examined alongside adaptive resistance mechanisms that may limit their clinical efficacy.

## Graphical Abstract

**Figure:**
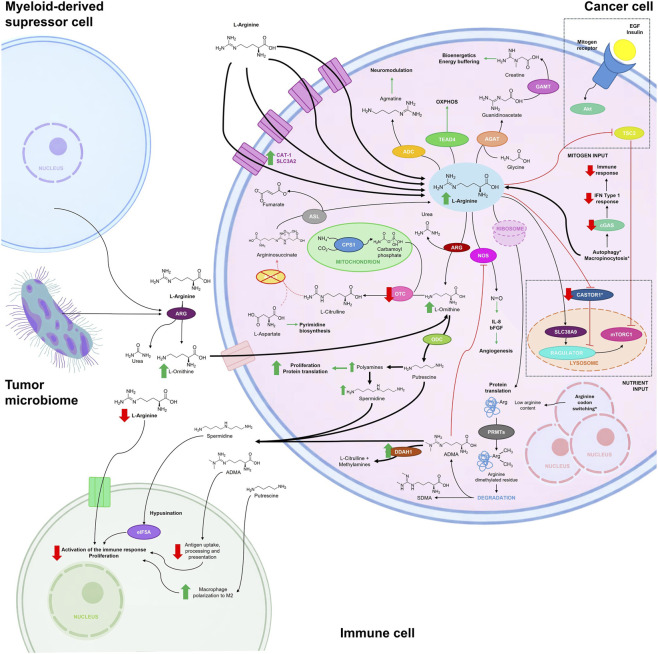

## Introduction

Cancer remains a major global health challenge, characterized by deregulated proliferation, aberrant differentiation, and disrupted tissue homeostasis driven by failure of growth-control networks. Its often asymptomatic progression leads to nearly half of cases being diagnosed at advanced stages, limiting therapeutic efficacy (Crosby et al., 2022). Thus, developing effective and accessible anticancer strategies remains a priority, requiring mechanistic insight into the shared principles governing malignant transformation and tumor persistence.

To systematize cancer complexity, the hallmarks framework was established and later expanded to encompass 14 capabilities acquired during tumorigenesis, defining core functions that drive malignant progression and guide therapeutic targeting (Hanahan, 2022). Within this systems-level view, L-arginine metabolism has emerged as a central regulatory axis linking multiple dimensions of tumor biology. Beyond a metabolic intermediate, L-arginine functions as a metabolic-immunologic hub, coordinating bioenergetics, biosynthesis, redox balance, and immune responses through its dual role as a substrate and signaling molecule modulating key nodes such as mTORC1.

Under physiological conditions, L-arginine homeostasis is tightly controlled through a coordinated network of extracellular transport, intracellular synthesis, and enzymatic conversion. L-Arginine is imported via high-affinity cationic amino acid transporters, including SLC7A1 (CAT-1), SLC7A2 (CAT-2), and SLC7A3 (CAT-3) (Banjarnahor et al., 2020), while endogenous synthesis from L-citrulline is mediated by enzymes of the urea cycle, such as carbamoyl phosphate synthase 1 (CPS1), ornithine transcarbamylase (OTC), and argininosuccinate synthase 1 (ASS1). This regulatory architecture confers metabolic flexibility, allowing non-malignant cells to maintain L-arginine availability even under conditions of nutrient fluctuation.

Beyond nitrogen metabolism, L-arginine functions as a key metabolic node whose conversion into polyamines, nitric oxide, agmatine, and creatine coordinates proliferation, chromatin remodeling, redox signaling, and energy buffering. Concurrently, intracellular L-arginine is sensed by proteins such as CASTOR1 and SLC38A9, which transmit amino acid sufficiency to the Rag GTPase-mTORC1 axis, linking nutrient availability to anabolic growth programs. This dual role underscores its function as an integrative regulator of cellular decision-making.

In cancer, this regulatory network is frequently rewired through coordinated alterations in L-arginine transport, biosynthesis, and utilization, redirecting flux toward pathways that sustain tumor growth, survival, and stress adaptation. A common outcome is functional L-arginine auxotrophy, whereby tumor cells lose endogenous synthesis capacity and depend on extracellular sources, revealing a targetable metabolic vulnerability.

In this mini-review, we move from a hallmark-based descriptive framework toward an integrative model positioning L-arginine as a metabolic-immunologic hub. By delineating how L-arginine metabolism orchestrates proliferation, stress responses, immune regulation, and microenvironmental adaptation, we provide a unifying perspective to inform more effective therapeutic strategies.

## Structural organization of the L-Arginine hub: metabolic flux and signaling integration

The functional relevance of L-arginine in cancer biology emerges from its unique position as a highly connected metabolic node, in which multiple biochemical pathways converge and diverge in a tightly regulated manner. This structural organization underpins its role as a metabolic-immunologic hub, capable of integrating nutrient sensing, biosynthetic demands, and signaling outputs.

At the core of this network, L-arginine is partitioned into distinct downstream pathways with specialized functions. Its hydrolysis to L-ornithine fuels polyamine biosynthesis via ornithine decarboxylase (ODC), supporting translation, chromatin organization, and proliferation. Concurrently, L-arginine serves as the obligate substrate for nitric oxide (NO) synthesis through nitric oxide synthase (NOS), linking its availability to redox signaling, vascular dynamics, and immune responses. Additional fates include decarboxylation by arginine decarboxylase (ADC) to generate agmatine, and conversion to creatine via arginine:glycine amidinotransferase (AGAT) and guanidinoacetate N-methyltransferase (GAMT), supporting cellular energy buffering through the phosphocreatine system.

These metabolic branches do not operate in isolation but compete for a shared intracellular L-arginine pool, such that flux redistribution directly shapes cellular behavior. For example, increased arginase activity enhances polyamine synthesis while limiting nitric oxide production, shifting the balance between proliferative and immunomodulatory outputs. This competitive architecture underscores L-arginine metabolism as an integrated regulatory network rather than discrete reactions.

In parallel, L-arginine functions as a key signaling molecule linking nutrient availability to growth control. L-Arginine sufficiency is sensed by CASTOR1 and SLC38A9, which relay signals to the Rag GTPase-mTORC1 axis, coordinating anabolic programs with nutrient status (Rebsamen et al., 2015; Saxton et al., 2016). Convergence of growth factor signaling on TSC2, whose activity is suppressed by L-arginine, further promotes mTORC1 activation, integrating mitogenic and metabolic inputs in a context-dependent manner (Carroll et al., 2016). Additional tissue-specific L-arginine-sensing mechanisms have been described, underscoring the context-dependent integration of local metabolic demands with specialized signaling networks.

This dual role of L-arginine as a metabolic substrate and signaling regulator confers high system plasticity, enabling rapid coupling of metabolic flux and signaling to environmental cues. While this flexibility preserves homeostasis in normal cells, cancer co-opts it to sustain malignant phenotypes and optimize resource allocation.

## Oncogenic rewiring of the L-Arginine hub: from metabolic flexibility to dependency

Building upon its intrinsic organization as a highly interconnected metabolic and signaling node, the L-arginine network undergoes profound reconfiguration in cancer. While in non-malignant cells L-arginine metabolism is characterized by flexibility and redundancy, tumor cells frequently reshape this system in a manner that restricts adaptability while enhancing selective advantages under proliferative and microenvironmental pressures.

A key consequence of metabolic rewiring is functional L-arginine auxotrophy, whereby tumor cells lose endogenous synthesis capacity and rely on extracellular L-arginine. This phenotype arises from coordinated alterations in enzymes and transport systems governing L-arginine homeostasis and reflects broader flux redistribution within the L-arginine hub. Increased arginase expression, particularly arginase 2 (ARG2), diverts L-arginine toward L-ornithine and polyamine biosynthesis, supporting proliferative and biosynthetic demands.

For instance, coordinated upregulation of CAT-1 and ARG2, coupled with reduced OTC expression, has been reported in sarcomas and brain tumors, impairing endogenous L-arginine synthesis (Vardon et al., 2017). Similarly, SLC3A2 overexpression is linked to L-arginine auxotrophy and a shift toward oxidative phosphorylation (OXPHOS) (Ren et al., 2025), while reduced or absent ASS1 expression has observed in a subset of esophageal cancers (Knipper et al., 2025). Notably, tumor cells can sustain anabolic signaling under L-arginine limitation by modulating sensing machinery. Downregulation of CASTOR1, for example, maintains mTORC1 activity, decoupling growth signals from nutrient availability and promoting malignant progression (Li et al., 2021).

These adaptations transform the L-arginine hub from a balanced and responsive network into a biased system characterized by preferential flux distribution and altered signaling thresholds. The consequences of this transformation extend beyond metabolism, influencing cell fate decisions, stress responses, and interactions with the tumor microenvironment. Thus, oncogenic rewiring of the L-arginine hub establishes a metabolic configuration that simultaneously supports tumor growth and creates exploitable weaknesses. Understanding the mechanisms that govern this transition is essential for the rational design of therapeutic strategies.

## L-Arginine availability as a determinant of proliferation, stress responses, and cell fate

In tumors, L-arginine availability governs the balance between proliferation and cell death. L-Arginine sufficiency activates mTORC1, enhancing protein and lipid biosynthesis while suppressing autophagy. Concurrently, L-arginine-dependent signaling stabilizes oncogenic pathways, reinforcing proliferative output. For instance, L-arginine enhances proliferation in colorectal and hepatocellular cancer cells. Mechanistically, it binds BAG2, modulating its interaction with SAMD4B and stabilizing β-catenin by preventing its degradation, thereby sustaining proliferative signaling (Chen M.-Y. et al., 2025). These effects are reinforced by coordinated metabolic reprogramming. L-arginine increases urea cycle flux, impacting carbamoyl phosphate utilization and its linkage to *de novo* pyrimidine biosynthesis, thereby supporting nucleotide availability and replicative capacity in upper gastrointestinal cancer cells (Tanaka et al., 2025). Additionally, NO promotes phosphorylation and inactivation of retinoblastoma protein, relieving E2F repression and driving cell cycle progression in breast cancer models (Radisavljevic, 2004).

In contrast, L-arginine deprivation disrupts this coordinated state and elicits a multilayered stress response. Early activation of AMPK reflects energetic imbalance and suppresses mTORC1, while p53 signaling, via phosphorylation of p53 and induction of p21, enforces cell cycle arrest in colorectal cancer models (Lin et al., 2025). Concurrently, amino acid scarcity activates the integrated stress response, where GCN2 and PERK drive eIF2α phosphorylation and ATF4 induction, with signaling intensity dictating adaptation versus irreversible damage (Morrow et al., 2013).

When L-arginine deprivation is prolonged or exceeds cellular adaptive capacity, stress responses shift toward cell death, with the mode determined by context. Arginase-based strategies to deplete L-arginine can trigger canonical apoptosis (Chew et al., 2024). In contrast, ADI-PEG20-mediated depletion initially activates AMPK and ERK, promoting cytoprotective autophagy that delays death, but its inhibition accelerates cell death, often via caspase-independent mechanisms (Kim et al., 2009).

Sustained stress induces mitochondrial dysfunction, collapse of oxidative phosphorylation, and reactive oxygen species accumulation, culminating in nuclear envelope disruption and sequestration of nuclear DNA within autophagosomes, a process termed chromatophagy (Szlosarek, 2014). In mesenchymal tumors, prolonged autophagy-dependent cytostasis can shift to necroptosis upon autophagy inhibition, associated with reduced caspase-3, caspase-8, and cIAP1, and assembly of the RIP1/RIP3-dependent ripoptosome (Bean et al., 2016).

Beyond its immediate effects on proliferation and survival, L-arginine availability intersects with long-term programs such as cellular senescence. Although incompletely defined, senescent cells exhibit reduced intracellular L-arginine alongside upregulation of L-arginine-catabolizing enzymes, including iNOS, ARG, ADC and AGAT, indicating metabolic remodeling (Li R. et al., 2025). In endothelial senescence, altered transport (increased hCAT-2B and reduced y^+^LAT1) and ARG2 upregulation divert L-arginine from NO synthesis toward urea and L-ornithine, promoting dysfunction, an effect reversible by ARG2 inhibition (Scalera et al., 2009). Conversely, chronic L-arginine excess can induce a senescence-like phenotype via sustained mTORC1-S6K1 activation, driving ARG2 overexpression and eNOS uncoupling (Xiong et al., 2014).

These observations reinforce the concept that L-arginine functions as a central regulator of cellular behavior within the tumor context. Its availability is continuously integrated through metabolic and signaling networks to determine whether cells proliferate, arrest, adapt, or die.

## The L-Arginine hub in the tumor microenvironment

The regulatory influence of the L-arginine hub extends beyond tumor-intrinsic processes to the tumor microenvironment, where metabolic and signaling interactions shape disease progression. In this context, L-arginine availability and flux coordinate not only proliferation and survival but also angiogenesis, immune function, and adaptive stress responses, reinforcing its role as an integrative axis in cancer biology.

A key manifestation of L-arginine-dependent regulation in the tumor microenvironment is its role in angiogenesis. L-Arginine-derived NO drives vascular dynamics by promoting endothelial activation, vasodilation, and pro-angiogenic signaling. L-Arginine and L-citrulline supplementation enhance angiogenesis via increased eNOS activity and NO production (Warden et al., 2025), while NO bioavailability is tightly regulated by asymmetric dimethylarginine (ADMA) and its degradation by dimethylarginine dimethylaminohydrolase-1 (DDAH1). Notably, DDAH1 is upregulated in prostate cancer, increasing NO production and promoting secretion of pro-angiogenic factors such as basic fibroblast growth factor and IL-8 (Reddy et al., 2018). Conversely, ADI-mediated L-arginine depletion suppresses angiogenesis in CHO and HeLa models, likely by limiting NO synthesis, although contributions from ammonia generated during L-arginine catabolism could not be excluded (Park et al., 2003).

Beyond angiogenesis, L-arginine metabolic reprogramming supports tumor adaptation to acidic microenvironments linked to progression and invasiveness. Under acidic stress, suppression of ASS1 reshapes L-arginine metabolism to maintain intracellular pH and promote survival, while favoring migratory and invasive phenotypes (Silberman et al., 2019). Collectively, these adaptations highlight L-arginine metabolism as a key driver of stress tolerance and tumor aggressiveness.

Over the past two decades, L-arginine has emerged as a key metabolic regulator of innate and adaptive immune responses within the tumor microenvironment (Zhu et al., 2025). In innate immunity, supraphysiological L-arginine supplementation reduces tumor burden and enhances natural killer (NK) cell cytotoxicity in colon cancer xenograft models (Yeh et al., 2010). Additionally, its downstream metabolites further shape antitumor immunity. Spermidine suppresses dendritic cell activation and T-cell priming mediated by eIF5A hypusination and prevents the canonical lipopolysaccharide-induced reduction in OXPHOS, thereby restraining the glycolytic shift associated with pro-inflammatory DC activation (Meehan et al., 2025), while putrescine attenuates M1 macrophage polarization, promoting collagen deposition and tumor progression (Isaza Correa et al., 2014; Xu et al., 2025). L-Arginine-derived ADMA impairs antigen uptake and presentation by downregulating key processing machinery (e.g., MHC class I, MHC class II, TAP1, TAP2, ERp57, and CD80) and attenuating CD4^+^ and CD8^+^ T-cell activation (Li M. et al., 2025). In contrast, NO supports innate immune surveillance by promoting M1 polarization and tumoricidal activity (Isaza Correa et al., 2014; Xiong et al., 2016; Xu et al., 2025).

Within adaptive immunity, elevated extracellular L-arginine during naïve T-cell activation promotes central memory-like differentiation and enhances T-cell persistence. This is coupled to a metabolic shift from glycolysis toward OXPHOS and appears to involve L-arginine-sensitive transcriptional regulators such as BAZ1B, PSIP1, and TSN (Geiger et al., 2016). Consistently, iNOS expression in murine models correlates with a Th1 cytokine profile characterized by IFN-γ, IL-2, and IL-12 production.

Beyond immune-regulatory pathways, microbiota-driven modulation of L-arginine metabolism further shapes tumor immunity. Enhanced intestinal bacterial L-arginine catabolism promotes angiogenesis and M2-like macrophage polarization in murine colorectal cancer models, fostering a pro-tumorigenic niche (Xu et al., 2025). However, microbiota-induced iNOS activation may result in sustained NO production in colorectal lesions, with context-dependent effects on tumor progression (Xu et al., 2025).

Conversely, L-arginine depletion in the tumor microenvironment, driven by arginases from myeloid-derived suppressor cells or bacteria, skews cytokine signaling (e.g., IL-4 and IL-13) toward a Th2 phenotype, establishing immune tolerance (Fletcher et al., 2015; Isaza Correa et al., 2014). Arginase 1 (ARG1)-mediated depletion further induces autophagy, suppresses cGAS-STING signaling, and attenuates type I interferon responses, collectively dampening antitumor immunity (Lamsal et al., 2024). Concurrently, L-arginine hydrolysis to L-ornithine fuels polyamine biosynthesis, supporting tumor growth and immune evasion, as reflected by elevated polyamine levels and ODC overexpression in multiple cancers (Bedoya et al., 2014; Isaza Correa et al., 2014).

Together, these observations highlight that L-arginine metabolism within the tumor microenvironment does not operate as isolated pathways, but rather as a dynamically regulated network in which metabolic flux redistribution directly shapes immune polarization and tumor progression.

## Discussion

Recognition of L-arginine metabolism as a central metabolic-immunological hub has enabled therapeutic strategies targeting its vulnerabilities. L-Arginine deprivation therapy (ADT), using PEGylated enzymes such as ADI-PEG20 or recombinant arginases, depletes extracellular L-arginine and shows clinical activity in L-arginine-auxotrophic tumors, including hepatocellular carcinoma and subsets of breast, colorectal, and hematological malignancies, with reported benefits such as prolonged progression-free survival in ASS1-deficient mesothelioma (Szlosarek et al., 2017) and meaningful disease control rates of 42.9% in acute myeloid leukemia (Tsai et al., 2017).

Despite these outcomes, ADT efficacy is often limited by adaptive resistance reflecting the plasticity of L-arginine metabolism. A key mechanism is ASS1 re-expression, restoring endogenous synthesis of L-arginine from L-citrulline. This response is embedded within oncogenic signaling pathways, including Ras/PI3K/ERK activation, which stabilizes c-MYC and enables displacement of transcriptional repressors such as HIF-1α and DEC1 from the ASS1 promoter, in cooperation with Sp4 (Bowles et al., 2008; Kuo et al., 2021; Long et al., 2016; Tsai et al., 2009). Concurrently, tumors enhance L-arginine uptake (e.g., CAT-1) (Prudner et al., 2018), engage alternative nutrient acquisition pathways such as NRF3-driven micropinocytosis (Hirose et al., 2023), and sustain mTORC1 signaling. Additional adaptations, including codon-switching mutations that reduce dependence on L-arginine-charged tRNAs (Hsu et al., 2023), and shifts toward more aggressive phenotypes (Chen O. et al., 2025), further underscore the metabolic flexibility of this axis under therapeutic pressure.

These limitations underscore the need to move beyond L-arginine depletion toward broader modulation of the L-arginine hub. Targeting key enzymatic nodes has emerged as a complementary strategy (Table 1): ARG1 inhibition (e.g., CB-1158) reverses myeloid-driven immunosuppression and enhances CD8^+^ T cell and NK cell infiltration (Steggerda et al., 2017), while dual ARG1/ARG2 inhibition (e.g., OATD-02) restricts L-arginine catabolism in both tumor cells and the microenvironment (Grzybowski et al., 2022). Likewise, DDAH1 inhibition (e.g., DD1E5) elevates ADMA, reduces NO production, and suppresses angiogenic and proliferative signaling pathways, including iNOS, VEGF, HIF-1α, and c-MYC (Kami Reddy et al., 2019).

**TABLE 1:** Compounds targeting arginine metabolism and associated pathways in cancer.

Compound	Description	References
CB-1158	Inhibition of ARG1 by CB-1158 was associated with enhanced infiltration of T cells and natural killer (NK) cells), accompanied by increased expression of pro-inflammatory cytokines and interferon-responsive genes. Moreover, ARG1 blockade by CB-1158 promoted T-cell proliferation and reduced tumor growth, both as a monotherapy and in combination with adoptive T-cell transfer, NK-cell therapy, immune checkpoint blockade, or gemcitabine	Steggerda et al. (2017)
OATD-02	OATD-02 potently inhibited ARG1 (IC_50_ = 17 ± 2 nM) and ARG2 (IC_50_ = 34 ± 5 nM). As monotherapy, it reduced the growth of immune-infiltrated CT26 tumors. In CT26-bearing mice, OATD-02, epacadostat, and anti-PD-L1 produced partial tumor growth inhibition (TGI: 33%, 41%, and 33%, respectively). However, the triple combination (OATD-02 + epacadostat + anti-PD-L1) achieved 87% TGI, whereas epacadostat + anti-PD-L1 did not outperform monotherapy	Grzybowski et al. (2022)
DD1E5	DD1E5, a small-molecule competitive inhibitor of DDAH1 (Ki ≈ 2.05 μM), elevates intracellular ADMA and suppresses NO production. This leads to reduced tumor cell proliferation, downregulation of VEGF, HIF-1α, c-Myc, and iNOS, inhibition of angiogenic factor secretion (bFGF and IL-8), and suppression of xenograft tumor growth, highlighting its potential as an anti-angiogenic therapeutic strategy in cancers with elevated DDAH1 activity	Kami Reddy et al. (2019)
Metformin	Metformin suppressed the immunosuppressive activity of granulocytic myeloid-derived suppressor cells (G-MDSCs) by reducing ARG1 activity and reactive oxygen species production, thereby restoring T-cell proliferation and enhancing antitumor immune responses in tumor-bearing mice. Metformin acted as a competitive inhibitor of gut microbial agmatinases (e.g., *E. coli* EcAGM), with Ki values in the millimolar range relevant to intestinal drug concentrations, potentially increasing agmatine levels and altering host–microbiome metabolic signaling. Metformin directly bound to the catalytic site of CARM1, inhibiting its methyltransferase activity and reducing histone H3R17 dimethylation, which led to decreased transcription of gluconeogenic genes such as PEPCK, FBPase1, and G6Pase	Dhang et al. (2025), Tassoulas and Wackett (2024), Xu et al. (2019)
Buformin, Phenformin	Phenformin and buformin acted as potent competitive inhibitors of *E. coli* agmatinase, with Ki values of 0.6 and 0.1 mM, respectively. Inhibition of bacterial agmatinase is expected to increase agmatine availability, potentially perturbing host-microbiome signaling pathways	Tassoulas and Wackett (2024)
GC-7	Deoxyhypusine synthase inhibitor that blocks eIF5A hypusination, leading to suppression of TCA cycle metabolism and synergistic cytotoxicity with ADI-PEG20 in mesothelioma cells	Carpentier et al. (2025)
ESAr	ESAr is an estrogen-conjugated nitric oxide synthase-targeting molecule designed for ER-expressing melanoma cells. ESAr induced apoptosis, suppressed proliferation and angiogenesis, and promoted differentiation-like phenotypic changes in melanoma cells, ultimately reducing tumor aggressiveness and preventing tumor formation in pretreated melanoma models	Roy et al. (2011)
Quinacrine	Quinacrine exerted antitumor activity in peripheral T-cell lymphoma by disrupting SLC3A2-mediated arginine uptake through targeting SSRP1. This prevented SSRP1-JUNB-driven SLC3A2 transcription, reduced intracellular arginine levels, and suppressed arginine-dependent metabolic reprogramming, including oxidative phosphorylation. Quinacrine also showed therapeutic synergy with histone deacetylase inhibitors, supporting a potential combinatorial strategy for PTCL treatment	Ren et al. (2025)
Agmatine	Agmatine inhibited tumor growth in S180 sarcoma and B16 melanoma mouse models and reduced the proliferation of MCF-7 breast cancer cells *in vitro* in a dose-dependent manner. The antiproliferative effect occurred without detectable cytotoxicity and was reversed by putrescine, suggesting that agmatine limits tumor cell growth by decreasing the intracellular polyamine pool. Also, it exerted an antiproliferative, anti-migratory, anti-invasive, and pro-apoptotic effect in Caco-2 colon cancer cell lines	Tanoglu et al. (2026), Wang et al. (2005)
CKi	CKi, a covalent creatine kinase inhibitor, exhibited minimal effects on cellular viability but markedly suppressed migration and invasion across several glioblastoma models. Metabolic and transcriptomic analyses further indicated that CKi induces a synthetic vulnerability to glutathione depletion and ferroptosis, revealing a metabolic liability that can be therapeutically exploited	Katz et al. (2024)
Allicin	Allicin, a reactive sulfur species derived from garlic, potently inhibited human ODC with an IC_50_ of 11 nM. By comparison, the established ODC inhibitor difluoromethylornithine (DFMO) displayed a substantially higher IC_50_ of 252 µM *in vitro*, indicating that allicin is approximately 23,000-fold more potent on a molar basis. Consistent with this activity, allicin at 50 µM markedly reduced cell viability and induced apoptosis in neuroblastoma cell lines	Schultz et al. (2020)
L-Norvaline	L-norvaline, an ARG1 inhibitor, in combination with doxorubicin inhibited proliferation, migration, and invasion in the 4T1 breast cancer cell line at a concentration of 100 mM. Additionally, it suppressed tumor growth through immune-mediated mechanisms and enhanced the antitumor efficacy of ADI-PEG20. The combination increased tumor-infiltrating CD8^+^ T cells and dendritic cells while reducing immunosuppressive myeloid populations	Ye et al. (2023), Zhu et al. (2022)
Nor-NOHA	Nor-NOHA is an arginase inhibitor reported to target ARG2 that induces dose-dependent apoptosis in leukemic cells under hypoxic conditions, with minimal effects under normoxia. However, its target specificity remains uncertain, as ARG2-knockout cells retain sensitivity to nor-NOHA, suggesting potential off-target mechanisms contributing to its anticancer activity	Ng et al. (2018)

Beyond enzyme targeting, small molecules that modulate L-arginine-linked metabolic and signaling pathways have gained attention. Biguanides such as metformin, buformin, and phenformin have been proposed to interfere with L-arginine metabolism (Espinosa-Rodriguez et al., 2023). Metformin reduced ARG1 activity in myeloid-derived suppressor cells, inhibited agmatinase, and suppressed the protein arginine methyltransferase CARM1, linking L-arginine metabolism to epigenetic regulation, with more potent effects reported for buformin and phenformin (Tassoulas and Wackett, 2024; Xu et al., 2019; Dhang et al., 2025).

Within this integrative framework, L-arginine metabolism should be considered in close interplay with glycolysis and lactate metabolism. L-Arginine availability sustains glycolytic flux and lactate production, while lactate reinforces L-arginine catabolism by promoting HIF-1α-driven M2 polarization and ARG1 expression (Gong et al., 2026; Kremer et al., 2017). In this context, lactylation links metabolic state to gene regulation, supporting immune suppression and tumor adaptation. Collectively, this crosstalk underscores the need to study L-arginine metabolism within a broader metabolic network involving glucose and lactate.

Collectively, these findings position L-arginine metabolism as a central metabolic-immunologic hub integrating tumor growth, immune evasion, angiogenesis, and stress adaptation, while dynamically interfacing with glycolysis and lactate metabolism. This crosstalk, where L-arginine availability sustains glycolytic flux and lactate production, and lactate in turn reinforces L-arginine catabolism and immunosuppressive signaling, highlights the complexity of this network and its therapeutic targeting. The limited efficacy of single-node interventions such as CB-1158 underscores this challenge (Naing et al., 2024). Accordingly, effective strategies will require combinatorial approaches that disrupt multiple interconnected pathways, including L-arginine and glucose-lactate metabolism, while anticipating adaptive responses. Future progress will depend on defining context-specific tumor and microenvironmental dynamics to enable rational, systems-level therapeutic design.
